# Comparative Analysis of Lycorine in Wild Plant and Callus Culture Samples of *Hymenocallis littoralis* by HPLC-UV Method

**DOI:** 10.1155/2014/408306

**Published:** 2014-05-06

**Authors:** Sreeramanan Subramaniam, Jeevandran Sundarasekar, Geethaa Sahgal, Vikneswaran Murugaiyah

**Affiliations:** ^1^School of Biological Sciences, Universiti Sains Malaysia, 11800 Penang, Malaysia; ^2^AIMST University, Jalan Bedong, Semeling, 08100 Bedong, Kedah Darul Aman, Malaysia; ^3^Institute for Research in Molecular Medicine, Universiti Sains Malaysia, 11800 Penang, Malaysia; ^4^School of Pharmaceutical Sciences, Universiti Sains Malaysia, 11800 Penang, Malaysia

## Abstract

The *Hymenocallis littoralis*, an ornamental and medicinal plant, had been traditionally used for wound healing. In the present study, an analytical method using HPLC with ultraviolet detection was developed for the quantification of lycorine in the extracts of different parts of wild plant and tissue culture samples of *H. littoralis*. The separation was achieved using a reversed-phase column. The method was found to be accurate, repeatable, and sensitive for the quantification of minute amount of lycorine present in the samples. The highest lycorine content was found in the bulb extract (2.54 ± 0.02 **μ**g/mg) whereas the least was in the root extract (0.71 ± 0.02 **μ**g/mg) of the wild plants. Few callus culture samples had high content of lycorine, comparable to that of wild plants. The results showed that plant growth regulators, 2,4-dichlorophenoxyacetic acid (2,4-D) alone at 4.5 **μ**M (2.58 ± 0.38 **μ**g/mg) or a combination of 2,4-D at 9.00 **μ**M with 4.5 **μ**M of 6-benzylaminopurine (BAP), were the optimum concentrations for the production of high lycorine (2.45 ± 0.15 **μ**g/mg) content in callus culture. The present analytical method could be of value for routine quantification of lycorine in the tissue culture production and standardization of the raw material or extracts of *H. littoralis*.

## 1. Introduction

*Hymenocallis littoralis* (Amaryllidaceae), known locally as “Melong kecil,” is an ornamental and bulbous perennial herb. It has been traditionally used in Philippines as a vulnerary [1]. Plants in the Amaryllidaceae family were reported to contain alkaloids that are known to exhibit a wide range of pharmacological activities [2]. A number of alkaloids were isolated from the* Hymenocallis littoralis* such as lycorine, littoraline, hippeastrine, lycorenine, tazettine, pretazettine, macronine, homolycorine, lycoramine, vittatine, and haemanthamine [3]. These compounds were reported to possess various pharmacological effects such as antiviral, antiparasitic, anticancer, antibacterial, antioxidant, and wound healing [4–6].

Lycorine, a pyrrolophenanthridine alkaloid, is one the major alkaloids found in* H. littoralis* [2, 7]. It displays strong antiviral effect against poliovirus, measles, and herpes simplex type 1 viruses [8]. Besides, lycorine also possesses potent antiretroviral [9], antimitotic [10, 11], and cytotoxic activities [2, 12].

Different analytical techniques have been described for the qualitative and quantitative determination of alkaloids in both wild plant and* in vitro* culture of Amaryllidaceae including GC-MS [2, 11, 12], spectrophotometric [2], HPTLC [11, 13], and enzyme immunoassay [2]. Few HPLC methods coupled with various detection methods were described for determination of lycorine in Amaryllidaceae plants such as* Galanthus* species [7],* Leucojum aestivum, *and* Pancratium maritimum *[2] and* Sternbergia *species [14] as well as tissue culture of* Pancratium maritimum *[15],* Narcissus confusus* [10], and* Leucojum aestivum* [16].

*In vitro *propagation is an important tool for rapid multiplication of medicinal plants [17, 18] as well as for the production of secondary metabolites. Tissue culture will ensure that the sources of the medicinal plants will not be exhausted or overexploited for their secondary metabolites. This is because the number of wild plants will not be effected due to overharvesting of the respective plants. Moreover the medicinal properties of a plant can be retained or increased via* in vitro* techniques. Previously, Yew et al. [18] have reported the effect of different cytokinins on* in vitro *shoot length and multiplication of* H. littoralis. *By adjusting phytohormones concentrations in the medium, differences in amount, rate, and growth patterns of explants were observed [19, 20].

Despite many publications on the pharmacological effects of its chemical constituents, there is very little information available on the phytochemical analysis of* H. littoralis* wild plants or callus culture. Likewise, there is no report on the capability of callus culture of* H. littoralis* to produce secondary metabolites such as lycorine which has important pharmacological effects. The establishment and quantification of lycorine via* in vitro* propagation technique could be a first step in mass production of any desired secondary metabolites in pharmaceutical industries. Thus, in the present study, a simple HPLC with UV detection method was developed for phytochemical analysis of different parts of* H. littoralis* wild plants. The method was further extended for the quantification of lycorine in callus culture obtained from various combinations of 2,4-dichlorophenoxyacetic acid (2,4-D) and 6-benzylaminopurine concentrations.

## 2. Material and Methods

### 2.1. Standards and Chemicals

The standard lycorine was purchased from Sigma-Aldrich (USA). Analytical grade methanol used for the extraction and HPLC grade acetonitrile and methanol used for analysis were purchased from Merck (Darmstadt, Germany). 2,4-D and BAP were purchased from Sigma (St. Louis, USA).

### 2.2. Chromatographic Conditions

The HPLC analysis was carried out using Gilson HPLC System (USA) comprising of solvent delivery pump and ultraviolet (UV) detector connected to a Hitachi D-2500 Chromato-integrator (Tokyo, Japan) for data collection. An Eclipse Plus reversed-phase C-18 column (250 × 4.6 i.d. mm, 5 *μ*m particle size; Agilent, USA) was used for separation. The mobile phase used was acetonitrile-1% aqueous ammonium acetate buffer, pH 6.0 (15 : 85 v/v) at the flow rate of 1.0 mL/min. The sample injection volume was 20 *μ*L and the wavelength of the detector was set at 325 nm.

### 2.3. Preparation of Standard Solution

The stock solution of lycorine was prepared by dissolving accurately weighed standard in methanol to give the concentration of 100 *μ*g/mL. The stock solution was then diluted with methanol in twofold (50 *μ*g/mL) dilutions to obtain a series of working standard solutions. All working standard solutions were stored in freezer at −20°C prior to analysis.

### 2.4. Preparation of* H. littoralis* Plant Materials

For the* ex vitro H. littoralis,* plants were bought from a nearby nursery in Jalan Masjid Negeri, Pulau Pinang. The plant materials were washed thoroughly and separated into different parts, namely, root, bulb, stem, leaves, flower, and anther.

For the* in vitro *plant, callus culture samples were obtained via tissue culture method. To initiate callus culture, the meristematic tissues of the bulb were cultured in semisolid Murashige and Skoog [21] media. Various concentrations of 2,4-D alone (2.25, 4.50, 9.00, 13.50, 18.00, and 22.50 *μ*M) and also with combination of 4.50 *μ*M BAP and 2.25, 4.50, 9.00, 13.50, 18.00, and 22.50 *μ*M of 2,4-D were used for the initiation of callus. For each concentration, four explants were cultured on the semisolid MS medium in a single culture jar (59 mm × 66 mm). It was then repeated three times which made up to a total of 16 replicates (4 explants × 4 jars). Finally, the cultures were kept in culture room under dark condition at 25 ± 2°C. Developed callus was collected after four weeks and dried in the oven at 50°C.

### 2.5. Extraction of Plant Materials

As for the* ex vitro* plant, the plant parts were dried in oven at 50°C until a constant weight was achieved and then were ground. The powdered plants were then extracted by sonication for 10 min at room temperature of 25 ± 2°C using methanol as the solvent (plant materials-solvent ratio of 1 : 5 to 1 : 10). After filtration, the filtrates were dried using a rotary evaporator (Buchi, Switzerland) at 40°C to obtain the respective extracts. The extracts were then kept in freezer at −20°C prior to analysis. Meanwhile, for* in vitro* plants, dried callus was then powdered using a mortar and pestle and extracted by sonication method as described above.

### 2.6. Validation of HPLC Method

#### 2.6.1. Limit of Detection (LOD), Limit of Quantification (LOQ), and Linearity

For the LOD and LOQ determination, the working standard solutions at different concentrations were injected into HPLC from the highest to the lowest concentrations. The concentration with the smallest detectable peak, at a noise-to-signal ratio of 3, was taken as the limit of detection (LOD), whereas the lowest concentration with acceptable accuracy and precision at noise-to-signal ratio of 8 was taken as the limit of quantification (LOQ). The linearity of the calibration curve was evaluated by linear regression analysis.

#### 2.6.2. HPLC-UV Method Accuracy and Precision

Validation of the HPLC method was carried out to determine the within-day and between-day accuracies and precisions. The standard stock solution was prepared by dissolving 1 mg of lycorine in 10 mL of methanol to produce a concentration of 100 *μ*g/mL. Series of working solutions were prepared by diluting the stock solution with methanol to give concentrations of 0.20, 0.78, 3.13, 12.50, and 50.00 *μ*g/mL. These working solutions were used to determine the within-day and between-day accuracies and precisions. The between-day analyses were carried out by injecting the standard working solutions once per day for five consecutive days while the within-day analyses were done by injecting the standard working solutions five times on the same day. A separate standard curve was constructed on each day of the validation study.

### 2.7. HPLC Analysis of* H. littoralis* Extracts

*H. littoralis* extracts analyses using HPLC-UV were carried out in two parts. The first part involved the quantification of the compound lycorine in methanolic extracts of different parts of* H. littoralis*, namely, root, bulb, stem, leaves, flower, and anther. The second part involved the quantification of the compound lycorine in callus extracts produced by using different combinations and concentrations of plant growth regulators (PGRs). Sample preparation for HPLC analysis was carried out by accurately weighing 2 mg of each dried extract, which was then dissolved in 2 mL of HPLC-grade methanol to give a concentration of 1 mg/mL. These samples were then filtered through a 0.45 *μ*m syringe filter (Whatman, Maidstone, England) and kept in fridge at −20°C prior to HPLC analysis.

### 2.8. Statistical Analysis

All experiments were repeated thrice with three replicates for each extract. The data were analyzed using IBM SPSS Statistics 20 software. Analysis of variance (ANOVA) and the means which were compared using the Tukey HSD at 5% level of significance (*P* < 0.05) were used.

## 3. Results 

### 3.1. HPLC-UV Method for Analysis of Lycorine

To date, there is very little information available on the phytochemical analysis or standardization of* H. littoralis* extract, and to our best knowledge there is no HPLC method reported for the analysis of phytochemicals in* H. littoralis*. Therefore, an HPLC-UV method was developed and validated for quantification of lycorine in various* H. littoralis *extracts. Lycorine absorbed maximum UV wavelength at 325 nm, which was selected as detection wavelength. The wavelength selected differs from previous reports which ranged between 287 and 292 nm [2, 7, 14]. Lycorine was eluted in the present HPLC method within 5 min and the total run time was 10 min.

In search of optimal chromatographic conditions, different mobile phase compositions were investigated. The mobile phases were acetonitrile-deionised distilled water (ddH_2_O) at ratios of 40 : 60 v/v, 35 : 75 v/v, 30 : 70 v/v, 25 : 75 v/v, 20 : 80 v/v, and 15 : 85 v/v, each at a flow rate of 1 mL/min. All these mobile phases resulted in peak splitting and tailing. The retention time was longer than 5 min. This phenomenon may be due to the ionization of the compound in the column.

Taking this into consideration, acetic acid or formic acid was added into the mobile phase to suppress the ionization of the compounds. Different concentrations of acetic acid ranging from 0.25 to 1% and formic acid ranging from 0.25 to 0.5% were added into the mobile phase. In addition, the aqueous phase was replaced with 10 mM of phosphate buffer or 1% ammonium acetate buffer. It was found that 1% ammonium acetate buffer without any addition of acetic acid or formic acid was the optimum mobile phase for separation of lycorine. Therefore, the mobile phase chosen for the subsequent analysis was acetonitrile-1% ammonium acetate (15 : 85 v/v).

**Figure 1:**
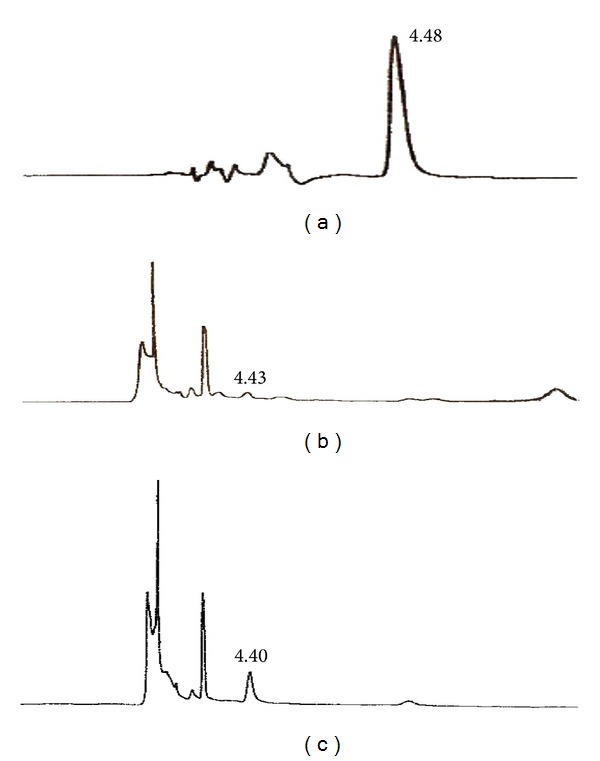
Chromatograms from the analysis of lycorine in* H. littoralis*. (a) Standard lycorine 6.25 *μ*g/mL. (b) Anther extract at 400 *μ*g/mL. (c) Extract of callus culture obtained with 4.5 *μ*M 2,4-D and 0 *μ*M BAP at 1000 *μ*g/mL. Column: Eclipse Plus reversed-phase C-18 (250 mm × 4.6 mm i.d., 5 *μ*m); mobile phase: acetonitrile-1% aqueous ammonium acetate buffer (15 : 85 v/v); flow rate: 1.0 mL/min; wavelength: 325 nm.

The HPLC chromatogram of marker compound, lycorine, is shown in Figure 1(a). The calibration curve of lycorine obtained with the developed HPLC method was linear over the range of concentration from 0.20 to 50 *μ*g/mL with a mean slope of 1493, mean intercept value of 177.8, and correlation coefficient of greater than 0.999. The within-day and between-day precisions and accuracies for the analysis of lycorine are shown in Table 1. The accuracy values, expressed as percentage of true value, were between 95.38% and 104.75%, while the corresponding precision values expressed as correlation of variation (CV) were between 0.73% and 8.15% for both within-day and between-day analysis, thus indicating that the present HPLC-UV method was reliable and repeatable.

The present HPLC-UV method had a lower LOQ compared to previous methods reported on analysis of lycorine, which were in the range of 1–34 *μ*g/mL [2, 7, 14]. The lower sensitivity of the present method permits the quantification of minute amount of lycorine present in the* ex vivo* plant tissues as well as the callus culture samples.

### 3.2. Quantification of Lycorine in* H. littoralis* Wild Plant and Tissue Culture Extracts

The developed method was used to quantify the amount of lycorine in extracts of various parts of the* H. littoralis* wild and tissue culture samples. Lycorine contents in those samples were calculated using external standard method and summarized in Table 2 while their representative chromatograms are shown in Figures 1(a) and 1(b). On the other hand, the contents of lycorine in extracts of callus samples are given in Table 3 while their representative chromatograms are shown in Figure 1(c). The wild plant extract and callus (initiated from 4.5 *μ*M 2,4-D alone and 9.00 *μ*M 2,4-D with 4.5 *μ*M BAP combination) were statistically analyzed. There was significant production of lycorine in callus compared to wild type plant extract.

## 4. Discussion

Generally, lycorine was detected in the extracts of all different parts of* H. littoralis*. As shown in Table 2, the highest content of lycorine was found in the bulb. This finding is in agreement with that of Abou-Donia et al. [13] that the bulbs of* Narcissus cv.* Breath of Spring plants possess higher amount of lycorine. Nevertheless, flower and anther extract also contained high amount of lycorine of 2.43 and 2.13 *μ*g per mg of extract, respectively. Stem and leaves extracts of* H. littoralis* had lesser lycorine content compared to previous plant parts, while the least amount of lycorine was found in the root extract.

Analysis of extracts of callus culture of* H. littoralis* revealed some interesting findings. Callus cultures supplemented with growth regulators at various combinations and concentrations produced lycorine at varying concentrations. For the callus cultures supplemented with only 2,4-D, lycorine content in the extracts increased when the amount of 2,4-D was increased from 2.25 to 4.50 *μ*M. However, further increase of 2,4-D up to 22.5 *μ*M caused no further increase or even gradual decrease in lycorine content. Hence, lycorine content decreases at higher 2,4-D concentrations in callus culture. At 2.25 *μ*M of 2,4-D, the amount of lycorine in the extract of callus culture was similar to that found in wild plant.

Combination of 4.5 *μ*M of BAP with various concentrations of 2,4-D affected the lycorine content of the callus extracts compared to those without BAP. At lower concentration of 2,4-D (2.25 *μ*M), addition of BAP greatly reduced the lycorine content. Similar finding was obtained for extracts of callus culture supplemented with 13.5 *μ*M of 2,4-D. In contrast, inclusion of BAP with 9.0 *μ*M, 18.0 *μ*M, or 22.50 *μ*M increased the lycorine content of 1.6 to 8.4 times higher compared to those callus cultures without BAP. However, when both agents were present at 4.5 *μ*M, the content of lycorine was similar to those callus extracts supplemented with only 2,4-D.

Comparing the amount of lycorine obtained from both wild plant and the callus tissue culture, it can be noticed that callus culture supplemented with appropriate concentrations and combination of growth regulators resulted in production of comparable amount of lycorine. This may be due to the effect of plant growth regulators (PGRs) such as the auxin and cytokinin used herein. PGRs are known to have effects not only on cell differentiation and proliferation but also on the biosynthesis pathway of the cell [22, 23]. Therefore, in this study, there may be a probability that the 2,4-D and BAP used in the callus culture could have partly been involved in the biosynthesis of lycorine. From the present study, the callus culture may be supplemented with only 4.50 *μ*M 2,4-D or a combination of 9.00 *μ*M 2,4-D with 4.5 *μ*M BAP to obtain a good yield of lycorine. This study can be further extended to investigate other combinations of PGRs that may increase the lycorine content of the callus culture. The findings of such study would prove to be useful as there are many benefits of the lycorine to the medical and health sector.

An analytical method using HPLC with ultraviolet detection was developed for the quantification of lycorine in extracts of different parts of wild plant and tissue culture samples of* H. littoralis*. The method was found to be accurate and repeatable and sensitive for the quantification of minute amount of lycorine present in the samples. The highest lycorine content in the wild plants was found in the bulb extract, whereas the least was in the root extract. Callus culture samples of* H. littoralis* had comparable lycorine content to that of wild plants. The results showed that 4.5 *μ*M 2,4-D alone or a combination of 9.00 *μ*M with 4.5 *μ*M BAP was the optimum concentrations for the production of high lycorine content in callus culture.

Statistical analyses were conducted for the lycorine content in callus (initiated via 4.5 *μ*M 2,4-D alone, 9.00 *μ*M 2,4-D with combination of 4.5 *μ*M BAP) and* H. littoralis* wild plant part extracts. The investigation shows that lycorine production in initiated callus at 4.5 *μ*M 2,4-D alone and 9.00 *μ*M 2,4-D with 4.5 *μ*M BAP is not significantly different while there is significant difference when compared with wild plant extract (*P* > 0.05). This indicates that the callus formed from these 2 different plant growth regulators is having high amount of lycorine compared to wild type plant. Thus, for the upcoming study, this hormone combination could be a good start to initiate a callus from* H. littoralis* to obtain secondary metabolites. Hence, the present analytical method may be of value for routine quantification of lycorine in the callus culture production and standardization of the raw material or extracts of* H. littoralis*.

## Figures and Tables

**Table 1:** Within-day and between-day precision and accuracy values for lycorine obtained from HPLC-UV analysis.

Concentration (µg/mL)	Within-day		Between-day	
	Accuracy (% true value)	Precision CV %	Accuracy (% true value)	Precision CV %
50.00	100.68	1.34	99.95	1.07
12.50	103.68	0.73	103.97	4.77
3.13	102.06	3.40	104.75	2.89
0.78	102.34	7.15	100.32	0.81
0.20	95.38	8.15	97.86	1.95

**Table 2:** Content of lycorine (dry weight) in the extracts of different parts of *H. littoralis* wild plant.

Content of lycorine (µg of lycorine per mg of extract)					
Root	Bulb	Stem	Leaves	Flower	Anther
0.71 ± 0.02*	2.54 ± 0.02*	1.72 ± 0.07*	1.37 ± 0.08*	2.43 ± 0.17*	2.13 ± 0.35*

*There is significant differences (*P* > 0.05) when compared with 4.5 µM 2,4-D alone and 9.00 µM 2,4-D with 4.5 µM BAP. *N* = 3.

**Table 3:** Content of lycorine (dry weight) in the extracts of *H. littoralis* callus samples.

Growth Regulators	Content of lycorine (µg of lycorine per mg of extract)					
2,4-D (µM)	2.25	4.50	9.00	13.50	18.00	22.50
Lycorine	1.79 ± 0.12	2.58 ± 0.38*	1.03 ± 0.25	1.48 ± 0.08	0.28 ± 0.01	0.17 ± 0.01

	Content of lycorine (µg of lycorine per mg of extract)					

2,4-D (µM) and 4.5 µM BAP	2.25	4.50	9.00	13.50	18.00	22.50
Lycorine	0.10 ± 0.05	2.45 ± 0.15*	2.12 ± 0.17	0.43 ± 0.03	0.45 ± 0.02	1.42 ± 0.09

*Significantly no difference, *P* < 0.05. *N* = 3.
